# Recovery of Chitin from *Agaricus bisporus* Mushrooms: Influence of Extraction Parameters and Supercritical CO_2_ Treatment on Fresh Mushrooms and Production Residues

**DOI:** 10.3390/molecules30071479

**Published:** 2025-03-26

**Authors:** Cláudia F. Almeida, Ivan Amorim, Cláudia G. Silva, José Carlos B. Lopes, Yaidelin A. Manrique, Madalena M. Dias

**Affiliations:** 1 LSRE-LCM—Laboratory of Separation and Reaction Engineering–Laboratory of Catalysis and Materials, Faculty of Engineering, University of Porto, Rua Dr. Roberto Frias, 4200-465 Porto, Portugal; 2 LEPABE—Laboratory for Process Engineering, Environment, Biotechnology and Energy, Faculty of Engineering, University of Porto, Rua Dr. Roberto Frias, 4200-465 Porto, Portugal; 3 ALiCE—Associate Laboratory in Chemical Engineering, Faculty of Engineering, University of Porto, Rua Dr. Roberto Frias, 4200-465 Porto, Portugal

**Keywords:** *Agaricus bisporus*, bio-residues, chitin, deacetylation degree

## Abstract

Chitin and chitosan, versatile biopolymers extensively used in the food and cosmetic industries, are traditionally sourced from crustaceans. However, fungi such as *Agaricus bisporus* mushrooms present a sustainable, non-animal alternative. This study explored the potential of different *Agaricus bisporus* samples, including fresh mushrooms and production residues, as sources of chitin. Given that *Agaricus bisporus* mushrooms are also a rich source of ergosterol, the study additionally incorporated samples treated with supercritical carbon dioxide (scCO_2_). The effects of deproteinisation conditions—specifically the number of successive extractions, sodium hydroxide concentration, and extraction time—were evaluated for fresh mushroom samples in terms of alkali-insoluble matter, chitin yields, and the degree of deacetylation (*DD*), with the latter determined by Fourier-transform infrared spectroscopy. The results indicated that extraction time had no statistically significant impact on AIM or chitin yield, while the *DD* increased with prolonged extraction, plateauing after 60 min. Higher sodium hydroxide concentrations enhanced deacetylation, but adversely affected extraction yields. No significant differences in chitin’s *DD* were observed between fresh mushroom and production residue samples, regardless of scCO_2_ treatment. This study demonstrates the viability of recovering chitin from *Agaricus bisporus* mushroom bio-residues, including those treated with scCO_2_, offering a sustainable and eco-friendly alternative for chitin production.

## 1. Introduction

**Figure 1:**
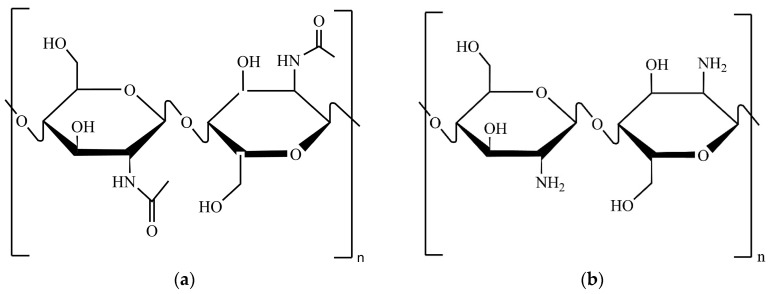
Chemical structures in the Haworth projection of: (**a**) chitin; (**b**) chitosan.

Chitin is the second-most abundant biopolymer in nature after cellulose and is synthesised by many organisms, including crustaceans (e.g., shrimp, crab, lobster, and squid), insects, algae, yeasts, and fungi [1,2]. Chitin and its derivative, chitosan, are polysaccharides composed of monomeric sugars linked by *O*-glycosidic bonds [2]. Their structures are similar to cellulose, except that they contain different functional groups in the C2 position of the glucose monomer unit (Figure 1). Chitin and chitosan consist of long chains of *N*-acetylglucosamine and d-glucosamine monomers, respectively, both linked by β(1 → 4) glycosidic bonds [3]. Chitin has drawn attention due to its health-promoting properties, including antioxidant, anti-inflammatory, antitumor and immune-stimulation activities [4]. Chitosan, recognised for its biocompatibility, biodegradability, and non-toxic nature, has also gained wide attention [5,6]. Its applications span various industries (e.g., food, paper, textiles, and cosmetics), and include the production of nanoparticles, membranes, microcapsules, pesticides, antimicrobial agents, superplasticisers, and coating materials [3,5,7,8,9]. However, intrinsic factors, such as the degree of deacetylation (DD), molecular weight (MW), and polydispersity (PD), influence the polymer’s physicochemical properties, thereby impacting its potential applications [9].

In industry, chitin is primarily extracted and isolated from shellfish waste (such as shrimp, crab, and lobster shells) through a process that requires harsh chemical reagents and high temperatures [4,6,7]. Commercial chitosan is then obtained by the deacetylation of chitin [10]. However, using seafood by-products for chitin recovery presents several limitations, including: seasonal and geographical supply restrictions; extraction of potential allergens; and difficulty in maintaining consistent composition and physicochemical properties of the final product [4,10]. These limitations have fueled interest in exploring alternative sources for chitin extraction.

Fungal sources offer numerous advantages: the use of a non-animal source; the production of chitin free from allergens such as tropomyosin, myosin light chain, and arginine kinase, which are commonly found in crustacean cuticles; milder extraction conditions due to lower mineral content, which consequently reduces chitin degradation and lowers costs; fewer extraction steps; and better control over the physicochemical properties of chitin, which can vary in crustaceans depending on the species and harvesting period [4,11,12,13]. Although commercial fungus-based chitin and chitosan products are currently limited, significant progress is being made in this direction [13]. For instance, the chitin–glucan (ChGC) complex extracted from *Aspergillus niger* has been approved as a marketed food additive. KiOnutrime-CsG^®^ (Kytozyme, Herstal, Belgium), a chitosan-based product, has achieved Generally Recognised as Safe (GRAS) status from the United States Food and Drug Administration (FDA) for use in beverages, is approved by the European Food Safety Authority (EFSA), and is registered under the novel food regulations in the European Union (EU). Chiber™, a mushroom-based chitosan product from the Canadian company Chinova Bioworks, is currently commercialised as a natural alternative to synthetic preservatives for food and beverages applications [12,13].

*Agaricus bisporus*, a mushroom species rich in chitinous biopolymers, is easy to grow and has a fast growth cycle, making it the most widely consumed species, accounting for over 38% of the global mushroom production [11,14]. However, mushroom harvesting and production generate waste equivalent to 20–30% of the total production volume, mainly consisting of discarded stalks or stipes, typically repurposed for low-economic-value uses such as animal feed and compost [15,16]. Thus, using *Agaricus bisporus* mushrooms, especially their production bio-residues, offers a promising opportunity for chitin recovery, as assessed in this study.

The recovery of chitin and chitosan from various fungal sources has been previously studied. John Kasongo et al. [1] reported the extraction of fungal chitin from *Termitomyces titanicus*, achieving a yield of 38.04%. The extracted chitin was subsequently deacetylated to produce chitosan. The deacetylation process was optimised using a full two-level factorial design considering the sodium hydroxide (NaOH) concentration and deacetylation time. The highest DD achieved was 69.50%, using a 50% NaOH concentration and a deacetylation time of 120 min. Papadaki et al. [4] determined the polysaccharide content of *Morchella* species mushrooms cultivated on various starch-based media, reporting yields ranging from 9.6% to 18.4% relative to the dry biomass. Erdogan et al. [5] extracted chitin from two mushroom species, obtaining 11.4% and 7.9% of chitin per dry weight of *Lactarius vellereus* and *Phyllophora ribis* mushrooms, respectively. Jones et al. [6] reviewed the differences between crustacean and fungal chitin, specifically regarding wound treatment. Wu et al. [7] quantified chitinous materials in *Agaricus bisporus* stalks during postharvest storage, observing an increase from 8.30% in fresh samples to 13.90% after 15 days at 4 °C and 27% after 5 days at 25 °C. Nitschke et al. [8] proposed a new method for quantifying chitin in edible mushrooms based on the reaction between polyiodide anions and chitosan and the resulting optical density of the insoluble polyiodide–chitosan complex. Their study reported higher chitin content in *Agaricus bisporus* mycelia (9.60%) compared to other mushroom species, namely *Pleurotus eryngii*, *Lentinula edodes*, *Morchella esculenta*, *Grifola frondosa*, *Pleurotus pulmonarius*, *Hypsizygus tessulatus*, *Trametes versicolor*, *Flammulina velutipes*, and *Pleurotus ostreatus*. Chitin content in *Agaricus bisporus* fruiting bodies was significantly lower, at 4.69%. Abdelghani Hassainia et al. [10] explored two routes for producing chitosan from *Agaricus bisporus* mushroom stipes. The first approach involved the direct deacetylation of mushroom powder, while the second method included the extraction of chitin followed by its deacetylation. The direct deacetylation approach resulted in a relatively low chitosan yield of 2.5%, whereas the chitin isolation method produced a significantly higher chitosan yield of 41%. In another study, A. Hassainia et al. [11] isolated chitin from different parts of *Agaricus bisporus*, obtaining the highest yield for stipes (7.4% on a dry weight basis) with a degree of acetylation (DA) of circa 70%. Boureghda et al. [12] investigated the extraction of the ChGC complex from *Pleurotus ostreatus* mushrooms, examining the effects of NaOH concentration, reaction time, and number of baths during the deproteinisation process. Additionally, the authors performed extractions from *Agaricus bisporus* stalks, reporting a chitinous material yield of 7%. Chou et al. [16] explored the potential of using polysaccharides derived from mushroom waste as prebiotics, reporting an enhanced survival rate of probiotics during cold storage and synergistic effects with amino acids and peptides from yogurt cultures to maintain high probiotic viability. Vetter [17] determined chitin content in different mushroom species, finding chitin content in *Agaricus bisporus* of 4.31% to 9.66% on a dry weight basis, higher levels than *Pleurotus ostreatus* and *Lentinula edodes*. Singh and Dutta [18] extracted chitinous material from *Agaricus bisporus* mushrooms, achieving a chitin yield of 1.2% (dry weight basis), and evaluated the antibacterial properties of the resulting ChGC complex. The chitin yield was found to be 1.2% based on dry weight. Nawawi et al. [19] and Mat Zin et al. [20] compared the morphological, mechanical, chemical, and surface properties of fungal chitin nanopapers with those of crustacean chitin.

This work focused on the recovery of fungal chitin from *Agaricus bisporus* mushrooms. First, the effect of various deproteinisation parameters on the extraction yield, $Y_{ext}$, and chitin’s degree of deacetylation, DD, was studied. These parameters included the number of successive extractions, reactant concentration, and extraction time. Subsequently, the influence of different *Agaricus bisporus* mushroom samples on $Y_{ext}$ and DD was evaluated. Fresh store-bought mushrooms and mushroom production residues were considered.

In our previous work [21], ergosterol was successfully recovered from *Agaricus bisporus* mushrooms using supercritical fluid extraction with carbon dioxide. This bioactive compound is recognised for its wide range of health benefits, including antioxidant, antitumor, anti-inflammatory, and antihyperlipidemic actions, and can also be converted into vitamin D_2_ through ultraviolet treatment [22,23,24,25]. Within the framework of a biorefinery concept, in this work, both fresh store-bought mushrooms and mushroom production residues post-extraction with supercritical carbon dioxide were also tested for chitin recovery.

## 2. Results and Discussion

### 2.1. Mushroom Characterisation

**Figure 2:**
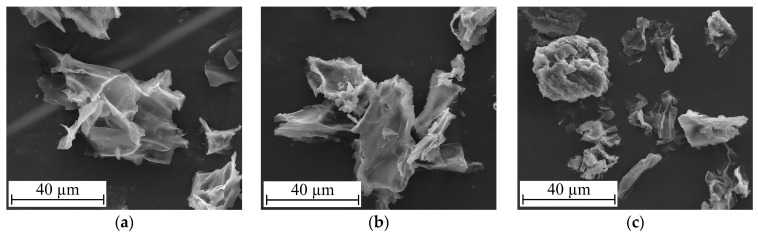
SEM images of different mushroom powder samples: (**a**) fresh; (**b**) fresh after scCO_2_ extraction; (**c**) production residue. All images were captured at 15 kV voltage with a working distance of 10.1 mm and a magnification of ×2500.

The morphology of different *Agaricus bisporus* mushroom samples was analysed by SEM. Figure 2 shows images of the fresh mushroom powder (both before and after scCO_2_ treatment) and of the mushroom production residue. All images were obtained under the same operating conditions, specifically at a voltage of 15 kV, a working distance of 10.1 mm, and a magnification of ×2500. Figure 2a,b show identical images for the fresh mushroom powder before and after scCO_2_ extraction, suggesting that this procedure does not affect the mushroom’s structure. Both images reveal porous structures, likely due to the mushrooms’ structural network being disrupted by their high water content and the formation of ice crystals during the freeze-drying stage. This observation is consistent with those of A. Hassainia et al. [11], who reported that the reagents could easily access the internal structure of the mushrooms, thereby favouring deproteinisation and deacetylation. On the other hand, the mushroom production residue samples (Figure 2c) display a slightly different morphology, possibly explained by the drying process to which they were subjected (oven-dried instead of freeze-dried). All images in Figure 2 show seemingly disrupted membranes, likely a result of the mushrooms’ grinding process.

**Figure 3:**
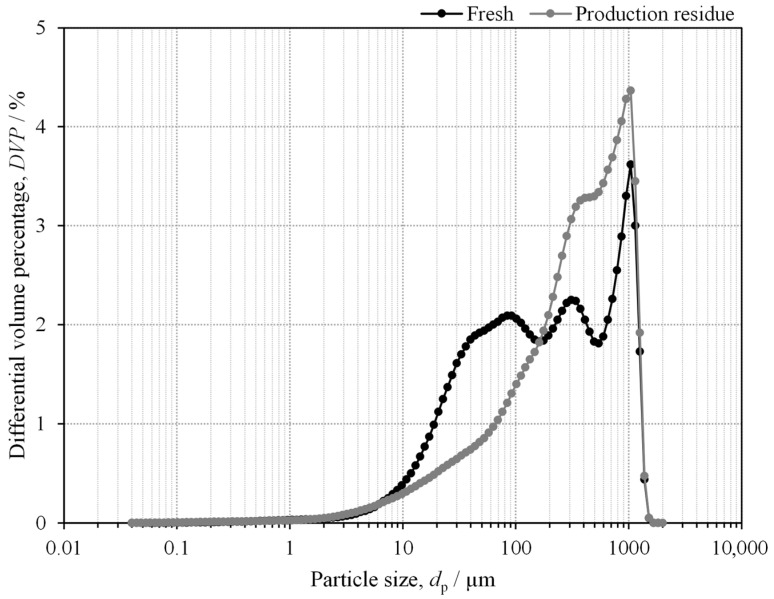
Volumetric particle size distribution of fresh (black) and production residue (grey) *Agaricus bisporus* mushroom powder. The x-axis is shown on a logarithmic scale for increased clarity.

Figure 3 shows the volumetric particle size distribution for the fresh (black) and production residue (grey) mushroom powders, presented in terms of differential volume percentage (DVP). A logarithmic scale is used on the x-axis to enhance perceptibility. The maximum value of the differential volume percentage occurs at the 1 mm channel for both samples. Furthermore, the mushroom production residue exhibits larger particles compared to the fresh mushroom powder, with average diameters of 359.9 µm for the fresh mushroom powder and 462.8 µm for the production residue mushroom powder. The volume percentage of fresh mushroom particles is higher than that of the production residue up to a particle size of 161.2 µm, but this trend reverses for larger particles. It is important to note that the size distribution calculations assume spherical particles, which is not confirmed in the SEM images of Figure 2, especially for the fresh mushroom. However, the morphology and size distributions indicate that the fresh mushroom powder has a porous structure and overall smaller particles (resulting in a higher interfacial area) compared to the production residue mushroom powder. Both factors are expected to enhance the efficiency of the deproteinisation and acid hydrolysis steps.

### 2.2. Influence of Deproteinisation Operating Conditions

The impact of deproteinisation parameters on both the AIM extraction yield ($Y_{ext}^{AIM}$) and chitin extraction yield ($Y_{ext}^{chitin}$), as well as on chitin’s degree of deacetylation (DD), was evaluated. Table 1 summarises the experiments performed, detailing the deproteinisation conditions and results obtained. Each experiment was performed in duplicate, with data presented as means ± standard deviation. Tukey’s honest significant difference (HSD) test at a 95% confidence interval was used to identify homogeneous groups for each dependent variable ($Y_{ext}^{AIM}$, $Y_{ext}^{chitin}$, and DD). The results are presented using a compact letter display, where mean values sharing a common letter are not significantly different, while those with different letters indicate statistically significant differences. This analysis encompassed all experiments (E01 through E13). However, results are divided into two tables: Table 1 presents data for experiments E01–E09, while Table 2 provides results for E10–E13. Consequently, the assigned letters may not follow a sequential order within each table, as they reflect the overall comparison across all experiments.

Three deproteinisation parameters were studied: deproteinisation time (*t*_DP_), assessed through experiments E01 to E04; number of successive deproteinisation steps (*n*), evaluated in experiments E04 to E07; and sodium hydroxide concentration (*C*_NaOH_), tested in experiments E04, E08, and E09. The individual effects of these parameters on *Y*_ext_ and *DD* are discussed in the following sections. Note that different y-axis scales are used in the *DD* plots for improved visibility.

#### 2.2.1. Deproteinisation Time

**Figure 4:**
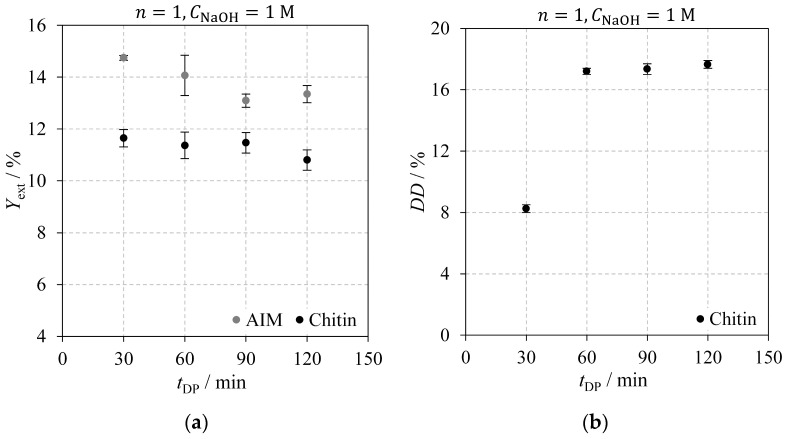
Impact of deproteinisation time on: (**a**) AIM (grey) and chitin (black) extraction yields; (**b**) chitin degree of deacetylation. Experiments were performed using a sodium hydroxide concentration of 1 M and a single deproteinisation step with fresh *Agaricus bisporus* mushroom powder.

Figure 4 shows the effect of deproteinisation time on the AIM and chitin extraction yields (Figure 4a) and on chitin’s DD (Figure 4b). Data shown in Figure 4 were obtained from experiments E01 through E04, conducted with a single deproteinisation step on fresh *Agaricus bisporus* mushrooms using a NaOH concentration of 1 M. Figure 4a shows nearly constant extraction yields with an apparent slight decrease at longer deproteinisation times. The AIM extraction yield drops from 14.7% at 30 min to approximately 13% for extractions lasting 90 min or more, while chitin’s extraction yield decreases from 11.7% for a 30 min extraction to 10.8% at 120 min. However, these changes were not statistically significant, as determined by Tukey’s HSD test at a 95% confidence interval. In Figure 4b, chitin’s degree of deacetylation rises with extraction time up to 60 min, beyond which the increase becomes less pronounced, stabilising around 17%. Tukey’s HSD tests revealed no statistically significant differences in DD among experiments E02–E04 for $t_{DP}\geq$ 60 min. Based on these results, the deproteinisation time was set to 120 min for subsequent tests.

#### 2.2.2. Number of Successive Deproteinisation Steps

**Figure 5:**
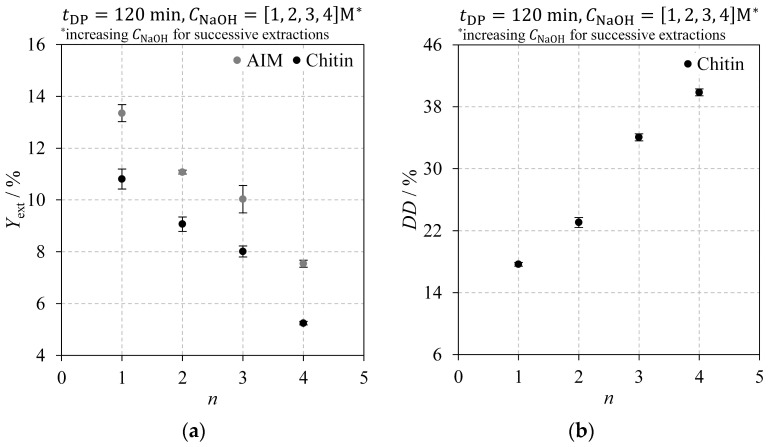
Impact of the number of successive deproteinisation steps on: (**a**) AIM (grey) and chitin (black) extraction yields; (**b**) chitin degree of deacetylation. Successive deproteinisation steps were performed using progressively increasing sodium hydroxide concentrations, with each step lasting 120 min on fresh *Agaricus bisporus* mushroom powder.

Figure 5 shows the impact of successive 120 min extractions on the AIM and chitin extraction yields (Figure 5a) and on chitin’s DD (Figure 5b). The experimental data in Figure 5 correspond to experiments E04 through E07, performed on fresh *Agaricus bisporus* mushrooms. Sodium hydroxide concentration was incrementally increased for consecutive extraction steps; for instance, for n=3, three consecutive 120 min deproteinisation steps were conducted with NaOH solutions at 1 M, 2 M, and 3 M. The results show that increasing the number of deproteinisation steps with higher NaOH concentrations reduces the AIM and chitin extraction yields (Figure 5a) while increasing chitin’s DD (Figure 5b). Significant differences among experiments were confirmed using Tukey’s HSD test, except for AIM and chitin extraction yields for experiments E05 and E06, which did not show statistically significant differences. The reduction in extraction yields likely reflects the cumulative effect of several factors: enhanced compound removal due to successive deproteinisation steps with fresh NaOH solutions; chitin’s deacetylation and conversion to chitosan, which has a lower molecular weight, thus yielding a lower mass for the same molar quantity, and greater mass loss from the additional washing steps, including centrifugation and vacuum filtration. In contrast, the rise in chitin’s DD for increased n (Figure 5b) primarily results from the higher sodium hydroxide concentrations rather than the step count itself. This is evident when comparing chitin’s DD from experiments E07 (n=4) and E08 (n=1, with 4 M NaOH), where a higher DD was obtained for a single deproteinisation step in E08 (see Table 1). Extraction yields were, however, lower in E07 (with successive deproteinisation steps) than in E08 (single extraction with 4 M NaOH), likely due to cumulative mass losses and more effective compound removal with repeated NaOH additions.

#### 2.2.3. Sodium Hydroxide Concentration

**Figure 6:**
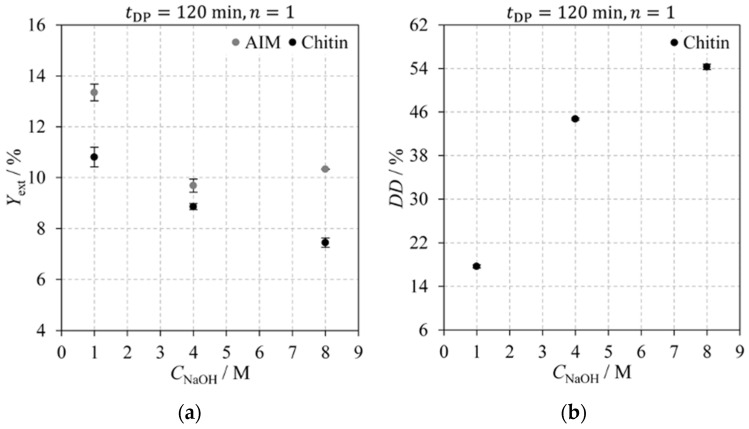
Impact of the sodium hydroxide concentration on: (**a**) AIM (grey) and chitin (black) extraction yields; (**b**) chitin degree of deacetylation. Experiments were performed for a single 120 min deproteinisation step on fresh *Agaricus bisporus* mushroom powder.

The impact of sodium hydroxide concentration on AIM and chitin extraction yields, and chitin’s DD is shown in Figure 6. This figure compiles data from experiments E04, E08, and E09, each performed using a single 120 min deproteinisation step on fresh *Agaricus bisporus* mushrooms. The results show that increasing sodium hydroxide concentrations lead to a decrease in extraction yields and an increase in chitin’s deacetylation degree. This reduction in extraction yields can be attributed to the conversion of chitin to chitosan and a potential enhancement in deproteinisation efficiency from using more concentrated solutions. An exception to this trend is observed in the AIM extraction yield for $C_{NaOH}=4\;M$: the difference between $Y_{ext}^{AIM}$ and $Y_{ext}^{chitin}$ is lower than all other experiments, and $Y_{ext}^{AIM}$ is lower than that at $C_{NaOH}=8\;M$. This result is probably an outlier, but due to limited sample availability, verification was not possible. These conclusions are supported by Tukey’s HSD test, which identified significant differences among most experiments, except for AIM and chitin extraction yields in experiments E08 and E09, where no statistically significant differences were observed.

In conclusion, the patterns observed in Figure 4b, Figure 5b, and Figure 6b indicate that NaOH concentration is the most influential factor affecting chitin’s degree of deacetylation.

### 2.3. Influence of Different Agaricus bisporus Samples

Table 2 compares the results for chitin extractions from different *Agaricus bisporus* mushroom samples, namely from fresh mushrooms and mushroom production residues, both before and after scCO_2_ extraction. These experiments were performed using a single 120 min deproteinisation step and $C_{NaOH}=1\;M$, except for experiment E11, where $C_{NaOH}=8\;M$. A compact letter display is used to represent homogeneous groups identified through Tukey’s HSD test at a 95% confidence interval. As previously noted, this analysis spans across all experiments (E01–E13), which accounts for the non-sequential assignment of letters.

There was no clear correlation between the type of mushroom sample and chitin’s degree of deacetylation (DD), as shown by experiments E04, E10, E12, and E13, and supported by Tukey’s HSD test. The DD variation among these experiments was minimal, at just 1.4%, indicating that the sample type—whether fresh, residue, or subjected to prior supercritical CO_2_ extraction—has a negligible effect on chitin’s deacetylation degree.

As expected, in all experiments, the value of $Y_{ext}^{chitin}$ was lower than $Y_{ext}^{AIM}$, as both were calculated relative to the initial sample mass. For the fresh mushroom experiments (E01 to E09, except E08), the average difference between $Y_{ext}^{AIM}$ and $Y_{ext}^{chitin}$ was circa 2.4%. However, samples that underwent supercritical CO_2_ extraction (E10 and E13) exhibited smaller differences: 0.5% for the fresh mushroom and 0.6% for the production residue. This reduced difference likely resulted from the extraction of certain compounds with the supercritical carbon dioxide, which would otherwise dissolve in the acetic acid solution.

Table 2 also indicates higher chitin extraction yields for mushroom production residue compared to fresh ones. Although this is an interesting observation, no definitive conclusion can be drawn, as the fresh mushrooms and the production residue were obtained from different sources Additionally, the different dehydration processes applied to the samples may also have contributed: fresh mushrooms were lyophilised, whereas production residues were oven-dried at 40 °C. This latter drying method may have caused the evaporation of volatile compounds, consequently increasing the relative chitin content in the mushroom matrix and resulting in higher extraction yields.

Finally, Table 2 shows experiment E11, performed on fresh *Agaricus bisporus* mushrooms after supercritical carbon dioxide extraction using a single 120 min deproteinisation step with an 8 M NaOH concentration. The results confirm previous conclusions, showing similar chitin extraction yields and deacetylation degrees when compared with experiment E09 (Table 1). No statistically significant differences were observed between experiments E09 and E11 for any of the dependent variables ($Y_{ext}^{AIM}$, $Y_{ext}^{chitin}$, and DD), as confirmed by Tukey’s HSD test.

### 2.4. Chitin Characterisation

**Figure 7:**
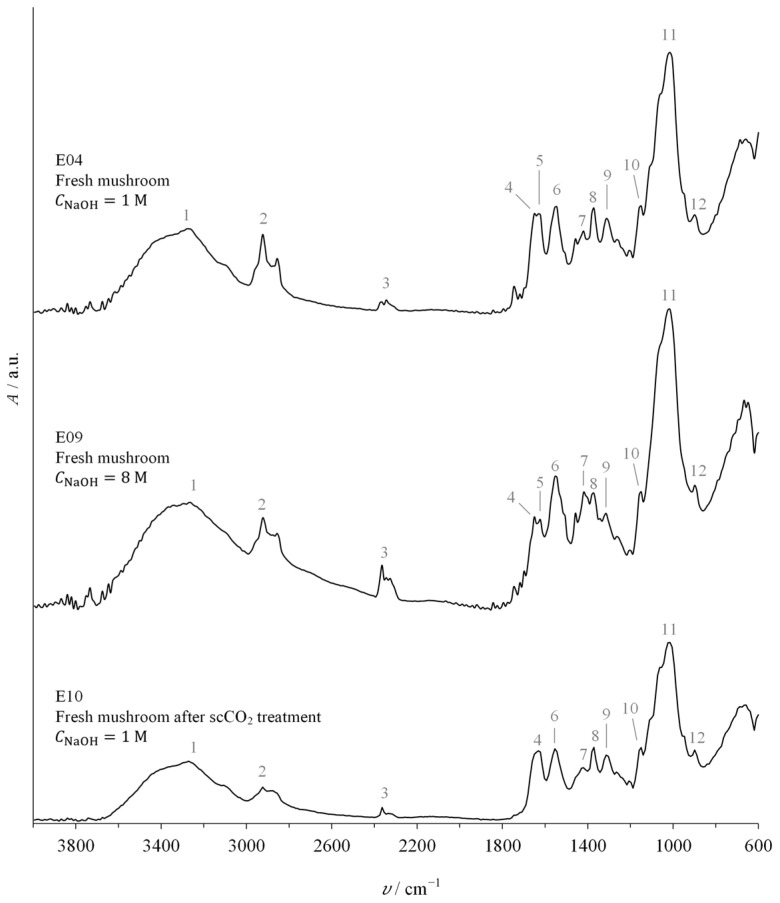
FTIR spectra of chitin extracted from fresh mushrooms: experiment E04, treated with $C_{NaOH}=1\;M$ (**top**); experiment E09, treated with $C_{NaOH}=8\;M$ (**middle**); and experiment E10, conducted on fresh mushrooms pretreated with scCO_2_ and with $C_{NaOH}=1\;M$ (**bottom**). All experiments were performed using a single 120 min deproteinisation step. Grey numbers identify peaks.

All chitin samples were analysed using FTIR spectroscopy, primarily to determine DD. This analysis can also be used to determine whether the deproteinisation process, particularly the concentration of NaOH, affects chitin quality. Figure 7 shows the FTIR spectra—absorbance (A) as a function of the wavenumber $\left(\upsilon \right)$—of chitin for experiments E04, E09, and E10. Experiments E04 and E09 differ in the NaOH concentration used for deproteinisation (Table 1), resulting in a DD=17.7±0.3% for E04 and DD=54.3±0.5% for E09, with a NaOH concentration eight times higher. Nevertheless, the spectra are very similar and closely resemble those reported by S. Dassanayake et al. [2] for chitin derived from shrimp shells, as well as by Liu et al. [26] for chitin extracted from shrimp and beetle (*Holotrichia parallela*). A split amide I band is clear at wavenumbers 1650 cm^−1^ and 1622 cm^−1^ in both spectra, indicating the presence of the α-chitin polymorph with anti-parallel alignment in crystalline zones [19,26,27]. The convoluted doublet appearance of the amide I band suggests a low-crystallinity structure [20].

Experiment E10, using fresh mushrooms after scCO_2_ extraction, also presents the typical chitin FTIR spectrum of experiment E04, conducted with fresh mushroom samples. A notable distinction between the two spectra is observed in the amide I band: E04 shows a split peak, indicative of α-chitin, while E10 exhibits a single peak, characteristic of β-chitin [27]. The scCO_2_ pretreatment may have induced a polymorphic transformation from α-chitin to β-chitin. Similar solvent-induced structural changes in chitin have been reported. For instance, Ramírez-Wong et al. [28] reported that exposure to deep eutectic solvents of different natures resulted in the formation of distinct crystalline phases of chitin films. A similar effect could have occurred with scCO_2_, particularly in the presence of ethanol as a polar modifier.

Furthermore, the absence of the 1540 cm^−1^ peak, associated with protein stretching vibrations, confirms the effectiveness of deproteinisation in all experiments, irrespective of sodium hydroxide concentration and material sample [11,29].

Table 3 summarises the main absorption peaks from Figure 7, detailing the associated bonds and their respective vibration modes. This data were compiled from several sources, including Sigma Aldrich [30], A. Hassainia et al. [11], Liu et al. [26], Majtan et al. [29], Cárdenas et al. [31], and S. Dassanayake et al. [2].

**Figure 8:**
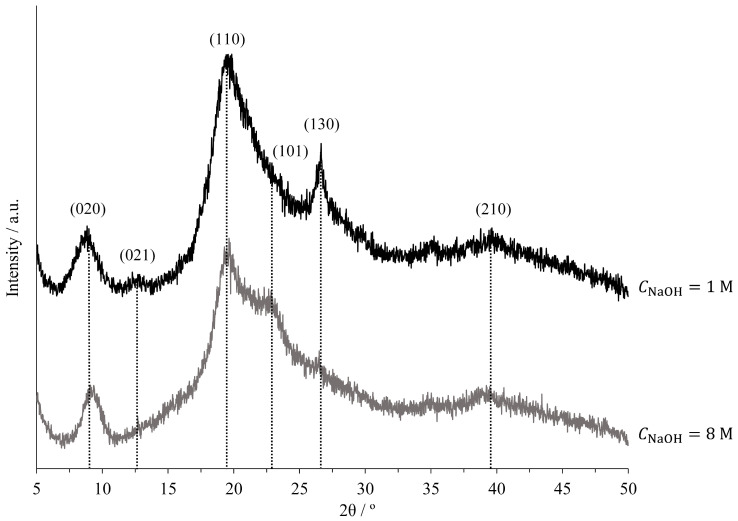
X-ray diffraction patterns of chitin extracted from fresh mushrooms: experiment E04, treated with $C_{NaOH}=1\;M$ (**top**); and experiment E09, treated with $C_{NaOH}=8\;M$ (**bottom**). Experiments were performed using a single 120 min deproteinisation step. Numbers indicate the family of planes according to Miller indices.

Additionally, selected chitin samples were characterised using XRD and TGA. Figure 8 shows the XRD patterns for chitin obtained from experiments E04 and E09, displaying the signal intensity (*I*) across various diffraction angles (2θ). The family of planes corresponding to the peaks is also indicated in the figure, along with their respective Miller indices (hkl) [11]. Table 4 summarises the information gathered from the XRD analysis, including chitin’s structural parameters, such as the family of planes according to Miller indices, peak diffraction angles, as well as the crystalline index (CI) and the crystallite size ($L_{hkl}$), calculated using Equations (3) and (4).

The XRD patterns shown in Figure 8 are characteristic of the α-polymorph, featuring two intense peaks at 9° and 19°, along with minor reflections at 12.8°, 22.8°, 26.6°, and 39° [2,20]. The absence of a peak at 2θ=6° in both samples indicates the effective removal of crystalline β-glucan [5,11,12]. In sample E04, the peak at 2θ=12.8° is attributed to residual protein components, as noted by Boureghda et al. [12]. Its absence in sample E09 is likely due to the more efficient removal of these compounds, facilitated by the eightfold increase in NaOH concentration during deproteinisation. The XRD patterns observed in Figure 8 are consistent with those reported in the literature for α-chitin extracted from insect, fungal, and crustacean sources [5,11,12,20,26,31,32]. As shown in Table 4, the crystalline index of chitin from E04 is higher than that from E09, which is expected since the CI decreases with increasing DD due to the deacetylation process [2,11]. The CI values obtained in this study align with those reported by other researchers. For example, Cárdenas et al. [31] estimated a CI between 76.2% and 82.7% for α-chitin extracted from various crustacean sources (shrimp, lobster, and crab); Liu et al. [26] reported a CI of 89% for both shrimp and *Holotrichia parallela* chitin; Erdogan et al. [5] found CI values of 64% and 49% for chitin extracted from the fungi *Lactifluus vellereus* and *Phyllophora ribis*, respectively; A. Hassainia et al. [11] calculated CIs of 88.1% and 63.2% for commercial α-chitin and chitin extracted from *Agaricus bisporus* stipes; Mat Zin et al. [20] produced chitin nanopapers with CIs ranging from 69.7% to 83.9%, derived from various sources (crab, shiitake, oyster, and enoki); and Triunfo et al. [32] obtained chitin from *Hermetia illucens* larvae, pupal exuviae, and dead adults, yielding a CI range of 62% to 96%.

**Figure 9:**
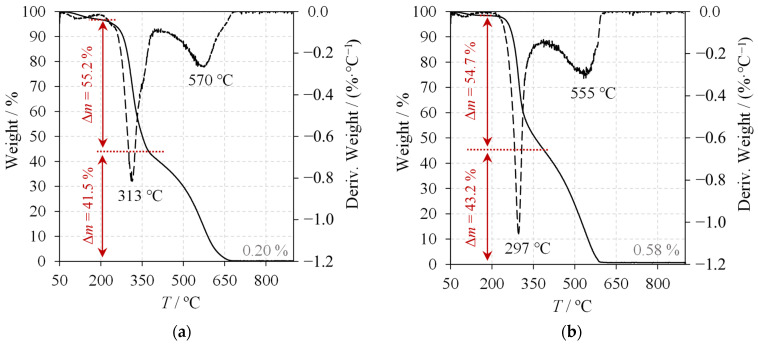
Thermograms (solid) and derivative thermogravimetric (dashed) curves: (**a**) chitin from experiment E04 with a DD=17.7%; (**b**) commercial chitosan with a DD=85%.

Figure 9 shows the thermograms of chitin from experiment E04 and a commercial chitosan sample (with a degree of deacetylation of 85%), along with the derivative thermogravimetric (DTG) curves represented by dashed lines. Figure 9 also shows the primary mass degradation stages, their corresponding temperatures, and the final ash content for each sample. Both thermograms exhibit similar behaviours, with mass degradation occurring in three main stages [11,12]. The first stage is attributed to the residual moisture content of the samples, resulting from the removal of physically adsorbed water [2]. During this initial stage, the mass loss was 1.53% for the chitin from E04 and 3.06% for the commercial chitosan. The second stage of mass degradation, occurring at 200–400 °C, corresponds to the degradation of the polymer chains, residual proteins, and glucans [11,12]. At this stage, the mass loss for E04’s chitin was 55.2% with a maximum DTG temperature of 313 °C compared to 54.7% mass loss and a 297 °C peak for the commercial chitosan. In the final stage, the degradation of the pyranose rings and the decomposition of residual carbon occur at temperatures between 400 and 650 °C [11,12]. The mass losses and maximum DTG temperatures for this stage were 41.5% and 570 °C for E04’s chitin compared to 43.2% and 555 °C for the commercial chitosan. Notably, these maximum DTG temperatures indicate that E04’s chitin exhibits higher thermal stability than commercial chitosan, as previously reported in the literature [2,5]. Previous studies have suggested that a lower DD favours the thermal stability of polymers and results in a higher CI, which was confirmed in this work [11,12]. Furthermore, Figure 9 shows that the ash content in E04’s chitin was lower than in commercial chitosan (0.20% versus 0.58%), indicating effective demineralisation during the autoclave acetic acid treatment [11,12].

## 3. Materials and Methods

### 3.1. Raw Material

**Figure 10:**
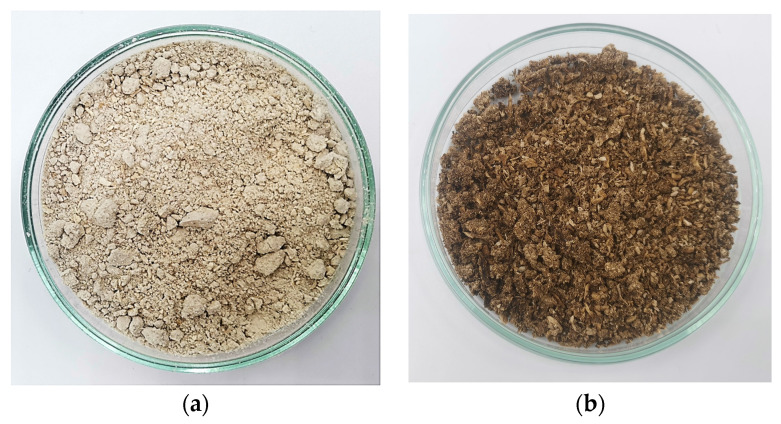
Mushroom powder used for chitin extraction: (**a**) fresh; (**b**) production residue.

*Agaricus bisporus* white mushrooms were used as a source for chitin extraction. In this study, the effect of four different mushroom samples on $Y_{ext}$ and DD was assessed. These samples included fresh mushrooms and production residues, both before and after extraction with supercritical carbon dioxide. Fresh mushrooms were purchased at a local store in March 2022. The samples were weighed, lyophilised in a VirTis benchtop K freeze-dryer for 48 h at a pressure of 40 mTorr and a temperature of −80 °C, and then reduced to a powder using a blender. The powder was stored in a ziplock bag, protected from light with aluminium foil, and placed in the freezer at −18 °C until further use. The water content was determined to be 92.37 ± 0.26% *w*/*w*. Mushroom production residues were sourced from a Portuguese mushroom production company and supplied by the Instituto Politécnico de Bragança (IPB). These residues were dried in an oven at 40 °C, accounting for the colour differences between samples in Figure 10. Additionally, scCO_2_-treated powders from fresh and mushroom production residue, which had previously been processed to recover ergosterol-enriched fractions [21], were also examined for chitin extraction. The samples were subjected to supercritical fluid extraction using carbon dioxide as the solvent. The extraction conditions were varied within the following ranges: temperature (40–60 °C), pressure (100–300 bar), and co-solvent volume percentage (0–10% *v*/*v*), with a constant flow rate of 4 mL/min (including both carbon dioxide and co-solvent). The extracts were recovered and analysed, while the spent mushroom powder was collected, homogenised, and subsequently used in the experiments described in this work.

### 3.2. Standards and Reagents

Sodium hydroxide standard solution (CAS 1310-73-2, 8 mol/L) was purchased from Fluka Analytical (Seelze, Germany); acetic acid glacial (CAS 64-19-7) was obtained from Fisher Chemical^TM^ (Waltham, MA, USA); and ammonium hydroxide solution (CAS 1336-21-6, ACS reagent) containing 28–30% NH_3_ basis was sourced from Sigma-Aldrich. Chitosan powder (CAS 9012-76-4, 85/10) was acquired from BioLog-Heppe GmbH (Landsberg, Germany). In this notation, the first number refers to the average deacetylation degree (85%), and the second number indicates the viscosity ($10\;\mathrm{mPa}\cdot s^{-1}$) for a 1% chitosan solution dissolved in 1% acetic acid. Deionised water was used to prepare all aqueous solutions and for the necessary washing steps.

### 3.3. Chitin Extraction

**Figure 11:**
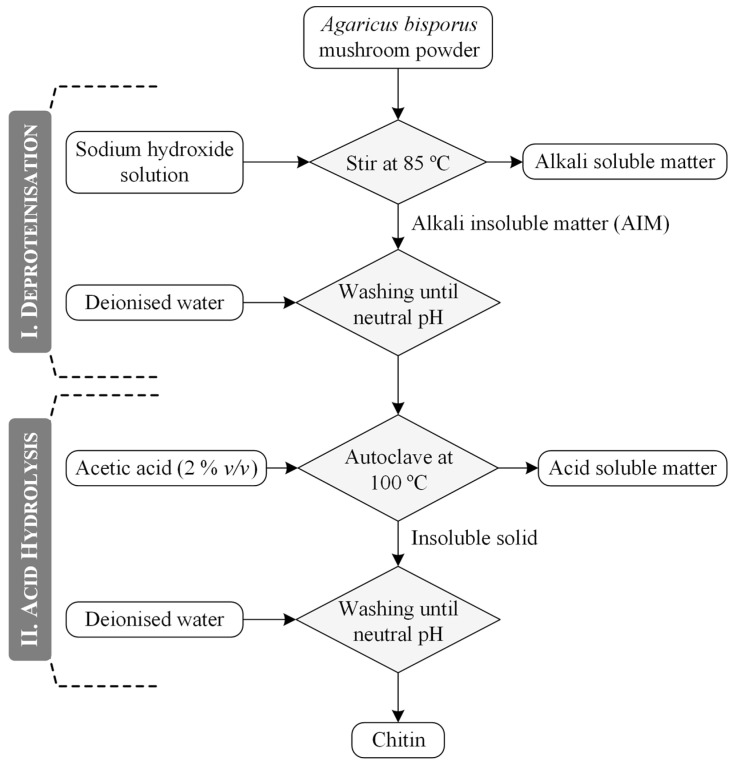
Schematic representation of the chitin extraction procedure.

The extraction of chitin from *Agaricus bisporus* mushrooms is shown schematically in Figure 11. This procedure, adapted from A. Hassainia et al. [11], comprises two main steps: an alkaline treatment for deproteinisation, followed by acid hydrolysis. First, the mushroom powder was treated with sodium hydroxide (NaOH) solution at a mass-to-volume ratio of 1:30 *w*/*v* and a temperature of 85 °C. This step removes proteins, alkali-soluble polysaccharides, and minor compounds such as monosaccharides, phenolics, amino acids, and salts [7]. The resulting insoluble solid, referred to as alkali-insoluble matter (AIM), was then subjected to multiple washing with deionised water until a neutral pH was achieved. Next, a 2% *v*/*v* acetic acid solution (100 mL) was added to the AIM, which was then placed inside an autoclave at 100 °C for 5 h. The extracted chitin, obtained as an insoluble solid, was thoroughly washed until reaching a neutral pH and then dried in an oven for yield quantification. Additionally, the filtrate resulting from the acid hydrolysis was collected and its pH increased (pH > 10) through drop-by-drop additions of a concentrated ammonium hydroxide solution (28–30% *v*/*v* NH_3_ basis). This procedure aimed to precipitate any chitosan present and was applied to all samples; however, the chitosan content was residual and therefore not further quantified.

Both the AIM and chitin were dried and weighed to calculate the extraction yield:

(1) $$Y_{ext}\left(\%\right)=\frac{m_{solid}\left(g\right)}{m_{dry\;mushroom}\left(g\right)}\times 100,$$

where $m_{solid}$ represents the mass of the extracted solid (either AIM or chitin) and $m_{dry\;mushroom}$ is the mass of the dry mushroom sample.

Table 5 summarises all the experiments performed, providing information on the raw material samples and deproteinisation conditions. Experiments E01 through E09, conducted with fresh *Agaricus bisporus* mushroom powder, examined the influence of various deproteinisation parameters, namely number of successive extractions (n), sodium hydroxide concentration ($C_{NaOH}$), and deproteinisation time ($t_{DP}$). E01 to E04 differ in the deproteinisation time, while experiments E04 to E07 vary in the number of successive extractions. In the latter cases, NaOH concentrations were progressively increased across successive 120 min extractions, indicated by an asterisk in Table 5. Furthermore, the effect of NaOH concentration for a single deproteinisation step was evaluated in experiments E04, E08, and E09. Due to the limited sample availability, subsequent experiments were carried out for a single 120 min deproteinisation step. In experiments E10 and E11, the effect of NaOH concentration was assessed for fresh *Agaricus bisporus* mushrooms after scCO_2_ treatment. Finally, experiments E12 and E13 were carried out with production residue samples before and after scCO_2_ treatment. All experiments were performed in duplicate. Statistical analyses were performed using TIBCO Statistica^®^ (version 14.0.0.15) software. One-way analysis of variance (ANOVA) and Tukey’s honest significant difference (HSD) test with a 95% confidence level (α=0.05) were used to identify statistically significant differences between groups. Homogeneous groups were determined based on the results of the post hoc test.

### 3.4. Characterisation Techniques

Samples of different *Agaricus bisporus* mushrooms were characterised in terms of morphology and powder size distribution using scanning electron microscopy (SEM) and laser diffraction analysis, respectively. The extracted chitinous polymers were analysed by Fourier-transform infrared spectroscopy (FTIR), thermogravimetric analysis (TGA), and X-ray powder diffractometry (XRD). A brief description of the characterisation methods is given next.

#### 3.4.1. Laser Diffraction Analysis

Volume-based size distributions of *Agaricus bisporus* mushroom samples were determined using a Beckman Coulter LS230 laser diffraction particle size analyser (Brea, CA, USA). Size distribution computations were based on the Fraunhofer model of light scattering, assuming spherical particles. Powdered fresh and production residue *Agaricus bisporus* mushrooms were dispersed in water for analysis. Each sample was measured twice, and the data presented are the average of both measurements.

#### 3.4.2. Scanning Electron Microscopy

SEM images were obtained using a high-resolution (Schottky) environmental scanning electron microscope equipped with X-ray microanalysis and electron backscattered diffraction analysis (FEI Quanta 400 FEG ESEM from Thermo Fisher Scientific, Waltham, MA, USA). Prior to observation, samples were coated with a thin gold–palladium (Au/Pd) film for 60 s at a 15 mA current using a SPI module sputter coater from Structure Probe Inc. Supplies (West Chester, PA, USA). The surface microstructure of the samples was observed at a voltage of 15 kV with a working distance (WD) of 10.1 mm using an Everhart–Thornley detector (ETD) to detect secondary electrons (SE). All images presented were taken at a magnification of ×2500.

#### 3.4.3. Fourier-Transform Infrared Spectroscopy

Fourier-transform infrared spectroscopy measurements were performed using a Nicolet 510-P spectrometer (ThermoFisher Scientific, Waltham, MA, USA), equipped with a MIRacle^TM^ single-reflection attenuated total reflectance (ATR) zinc selenide (ZnSe) crystal plate accessory (PIKE Technologies, Madison, WI, USA). Spectra were recorded over a wavenumber range of 600–4000 cm^−1^, with 256 scans and a resolution of 4 cm^−1^.

The degree of acetylation (DA) of chitin was estimated from the obtained spectra using the methodology proposed by Baxter et al. [33], which relates the infrared absorbances of the C=O amide bond and the hydroxyl group (O-H) according to:

(2) $$DA\left(\%\right)=115\frac{A_{1655}}{A_{3450}},$$

where $A_{1655}$ and $A_{3450}$ are the absorbances at wavenumbers (υ) 1655 cm^−1^ and 3450 cm^−1^, respectively. The degree of deacetylation (DD) is assumed as the complementary percentage of the DA, $DD\left(\%\right)=100-DA\left(\%\right)$.

#### 3.4.4. Thermogravimetry Analysis

Thermogravimetric analyses were carried out using a Netzsch STA 409 PC analyser (Selb, Germany). Approximately 10 mg of each sample was heated in an oxygen (O_2_) atmosphere from 50 °C to 900 °C at a heating rate of 10 °C$\cdot \min^{-1}$. Moisture and ash content were determined from the resulting thermograms.

#### 3.4.5. X-Ray Powder Diffractometry

X-ray diffraction analyses were performed using a Malvern PANalytical X’Pert Pro diffractometer (Almelo, The Netherlands) with CuKα radiation ($\lambda_{1}=1.54060$ Å, $\lambda_{2}=1.54443$ Å) equipped with an X’Celerator detector and a secondary monochromator in θ/2θ Bragg–Brentano geometry. The equipment was operated in a 2θ angular range of 5° to 60° at 40 kV and 30 mA. Scans were conducted with a step size of 0.017° and a step time of 120 s. These analyses allowed for the determination of chitin’s crystalline index (CI) and crystalline size.

The crystalline index was determined using the methodology proposed by Segal et al. [34] for cellulose and previously applied [5,11,12,26] to determine the CI of chitin:

(3) $$CI\left(\%\right)=\frac{I_{110}-I_{am}}{I_{110}}\times 100,$$

where $I_{110}$ is the diffraction intensity of the (110) plane at 2θ ≈ 19° and $I_{am}$ is the amorphous diffraction intensity at 2θ ≈ 16°. The crystalline size, $L_{hkl}$, in the perpendicular direction of the lattice planes, was calculated using Scherrer’s equation:

(4) $$L_{hkl}=\frac{K\lambda }{\beta \cos\theta },$$

where K is Scherrer’s constant, which is related to the crystallite’s shape and has a typical value of 0.9; λ is the X-ray wavelength (1.54060 Å); β is the full width at half maximum (FWHM) of the diffraction peak; and θ is the diffraction angle [11,35].

XRD data were processed using Origin Pro 2023 software, applying the Savitzky–Golay method for curve smoothing and the Gaussian function for peak deconvolution. The FWHM of the peaks was determined using the Peak Multiple Fit tool.

## 4. Conclusions

This work focused on the recovery of chitin from *Agaricus bisporus* mushrooms by testing different deproteinisation operating conditions, including time, sodium hydroxide concentration, and number of successive extractions. Among these factors, sodium hydroxide concentration emerged as the most influential, significantly affecting both the extraction yield and the degree of deacetylation of chitin. Specifically, higher NaOH concentrations led to lower extraction yields while increasing the DD of chitin; for instance, a 1 M sodium hydroxide solution resulted in chitin with a DD of 17.7 ± 0.3%, while an eightfold increase in concentration raised the DD to 54.3 ± 0.5%. These results suggest that further increases in the alkaline solution concentration, along with higher deproteinisation temperatures, may lead to the conversion of chitin into chitosan.

Additionally, this study tested various *Agaricus bisporus* samples, including fresh mushrooms and mushroom production residues—both before and after supercritical carbon dioxide extraction—under identical conditions of a single 120 min deproteinisation with a NaOH concentration of 1 M. Tukey’s HSD test showed no statistically significant differences in chitin’s DD between these samples, indicating that *Agaricus bisporus* residues are suitable and sustainable sources for chitin recovery. Furthermore, prior supercritical CO_2_ extraction of bioactives can be performed without causing significant differences in chitin yield or quality.

The findings of this study demonstrate the potential of *Agaricus bisporus* samples, including mushroom production residues treated with scCO_2_, as sustainable sources for chitin recovery. This approach provides a non-animal alternative to traditional sources and can be integrated into a biorefinery framework that recovers ergosterol-enriched fractions through supercritical fluid extraction, followed by chitin recovery from the spent mushroom residues. Future research should focus on the use of green extraction technologies to further enhance chitin recovery and reduce environmental impact. Additionally, assessing the scalability and economic feasibility of these methods is crucial for evaluating their practical applicability. The recovered chitin should also be tested for its potential use in bioplastics, pharmaceuticals, agriculture, and other sectors.

## Figures and Tables

**Table 1:** Impact of different deproteinisation conditions on AIM and chitin extraction yields, as well as chitin’s degree of deacetylation. Experiments were performed on fresh *Agaricus bisporus* mushroom samples.

ID	Material Sample	Deproteinisation			$Y_{ext}^{AIM}$/%	$Y_{ext}^{chitin}$/%	DD/%
		n	$C_{NaOH}/M$	$t_{DP}/min$			
E01	Fresh	1	1	30	14.7 ± 0.1 ^a^	11.7 ± 0.3 ^bc^	8.3 ± 0.3 ^g^
E02	Fresh	1	1	60	14.1 ± 0.8 ^a^	11.4 ± 0.5 ^c^	17.2 ± 0.2 ^f^
E03	Fresh	1	1	90	13.1 ± 0.3 ^ab^	11.5 ± 0.4 ^bc^	17.4 ± 0.4 ^f^
E04	Fresh	1	1	120	13.4 ± 0.3 ^ab^	10.8 ± 0.4 ^c^	17.7 ± 0.3 ^f^
E05	Fresh	2	1, and 2 *	120	11.1 ± 0.1 ^cd^	9.1 ± 0.3 ^d^	23.1 ± 0.7 ^e^
E06	Fresh	3	1, 2, and 3 *	120	10.0 ± 0.5 ^cde^	8.0 ± 0.2 ^def^	34.1 ± 0.5 ^d^
E07	Fresh	4	1, 2, 3, and 4 *	120	7.5 ± 0.1 ^f^	5.2 ± 0.1 ^g^	39.9 ± 0.5 ^c^
E08	Fresh	1	4	120	9.7 ± 0.3 ^de^	8.9 ± 0.1 ^de^	44.7 ± 0.2 ^b^
E09	Fresh	1	8	120	10.3 ± 0.01 ^cde^	7.5 ± 0.2 ^ef^	54.3 ± 0.5 ^a^

* Increasing NaOH concentration for successive 120 min extractions. Mean values sharing the same letter in the Tukey grouping are not significantly different (*p* > 0.05).

**Table 3:** Main infrared absorption peaks with their corresponding bonds and vibration modes.

Peak	υ/cm^−1^	Bond and Vibration Mode	Remarks
1	3500−3200	O-H and N-H stretching	Broad peak
2	3000−2800	C-H stretching	Symmetrical and asymmetrical vibrations of polysaccharides
3	2349	O=C=O stretching	Atmospheric carbon dioxide
4	1650	C=O stretching	Amide I
5	1622	C=O stretching	Amide I
6	1554	N-H bending and C-N stretching	Amide II
7	1429−1410	CH_2_ bending	-
8	1371	C-H and C-CH3 bending	-
9	1323−1304	C-N stretching and N-H bending	Amide III
10	1159	C-O-C stretching	Asymmetrical bridge of C-O-C (ring)
11	1014	C-O stretching	-
12	899	C-H	β-glycosidic bonds

**Table 2:** Impact of different *Agaricus bisporus* samples on AIM and chitin extraction yields, as well as on chitin’s degree of deacetylation. Experiments were performed for a single 120 min deproteinisation step.

ID	Material Sample	Deproteinisation			$Y_{ext}^{AIM}$/%	$Y_{ext}^{chitin}$/%	DD/%
		n	$C_{NaOH}/M$	$t_{DP}/min$			
E04	Fresh	1	1	120	13.4 ± 0.3 ^ab^	10.8 ± 0.4 ^c^	17.7 ± 0.3 ^f^
E10	Fresh after scCO_2_ extraction	1	1	120	11.6 ± 0.2 ^bc^	11.1 ± 0.2 ^c^	16.9 ± 0.4 ^f^
E11	Fresh after scCO_2_ extraction	1	8	120	8.7 ± 0.3 ^ef^	7.1 ± 0.03 ^f^	54.1 ± 0.2 ^a^
E12	Production residue	1	1	120	14.1 ± 0.3 ^a^	12.9 ± 0.2 ^ab^	17.6 ± 0.2 ^f^
E13	Production residue after scCO_2_ extraction	1	1	120	14.4 ± 0.1 ^a^	13.8 ± 0.1 ^a^	18.3 ± 0.1 ^f^

Mean values sharing the same letter in the Tukey grouping are not significantly different (*p* > 0.05).

**Table 4:** Chitin structural parameters and crystallinity index determined by X-ray diffraction.

ID	Plane	2θ/°	*L_hkl_*/Å	CI/%
E04	020	8.98	36.8	88.4
	021	12.84	28.3	
	110	19.42	14.9	
	130	26.64	19.3	
	210	39.63	23.9	
E09	020	9.34	33.8	78.4
	110	19.64	24.9	
	101	22.83	9.2	
	210	39.46	6.5	

**Table 5:** Summary of all experiments performed, including information on the raw material samples and the conditions used in the deproteinisation step.

ID	Raw Material Sample	Deproteinisation Parameter		
		n	$C_{NaOH}/M$	$t_{DP}/min$
E01	*Agaricus bisporus* fresh mushroom	1	1	30
E02	*Agaricus bisporus* fresh mushroom	1	1	60
E03	*Agaricus bisporus* fresh mushroom	1	1	90
E04	*Agaricus bisporus* fresh mushroom	1	1	120
E05	*Agaricus bisporus* fresh mushroom	2	1, and 2 *	120
E06	*Agaricus bisporus* fresh mushroom	3	1, 2, and 3 *	120
E07	*Agaricus bisporus* fresh mushroom	4	1, 2, 3, and 4 *	120
E08	*Agaricus bisporus* fresh mushroom	1	4	120
E09	*Agaricus bisporus* fresh mushroom	1	8	120
E10	*Agaricus bisporus* fresh mushroom after scCO_2_ extraction	1	1	120
E11	*Agaricus bisporus* fresh mushroom after scCO_2_ extraction	1	8	120
E12	*Agaricus bisporus* production residue mushroom	1	1	120
E13	*Agaricus bisporus* production residue mushroom after scCO_2_ extraction	1	1	120

* Increasing NaOH concentration for successive 120 min extractions.

## Data Availability

The dataset is available on request from the authors.

## Abbreviations

The following abbreviations are used in this manuscript:

AIM	alkali-insoluble matter
ANOVA	analysis of variance
ATR	attenuated total reflectance
ChGC	chitin–glucan
CI	crystalline index
DA	degree of acetylation
*DD*	degree of deacetylation
DTG	derivative thermogravimetric
DVP	differential volume percentage
EFSA	European Food Safety Authority
ETD	Everhart–Thornley detector
EU	European Union
FDA	Food and Drug Administration
FTIR	Fourier-transform infrared spectroscopy
FWHM	full width at half maximum
HDS	honest significant difference
IPB	Instituto Politécnico de Bragança
MW	molecular weight
PD	polydispersity
scCO₂	supercritical carbon dioxide
SE	secondary electrons
SEM	scanning electron microscopy
TGA	thermogravimetric analysis
WD	working distance
XRD	X-ray powder diffractometry
ZnSe	zinc selenide
